# A high occurrence of late presenters and missed HIV diagnosis in clinical care in Sweden

**DOI:** 10.1186/1758-2652-13-S4-P169

**Published:** 2010-11-08

**Authors:** J Brännström, V Svedhem, A Yilmaz, A Blaxhult, S Wendahl, A Sönnerborg

**Affiliations:** 1 Inst of Medicine, Karolinska Institute, Karolinska University Hospital, Department of Infectious Diseases, Stockholm, Sweden; 2 Institution of Medicine, Karolinska Institutet, Karolinska University Hospital, Department of Infectious Diseases, Stockholm, Sweden; 3 Sahlgrenska University Hospital, Department of Infectious Diseases, Gothenburg, Sweden; 4 Venhälsan, Södersjukhuset, Stockholm, Sweden; 5 Sunderby Hospital, Department of Infectious Diseases, Luleå, Sweden; 6 Inst of Medicine, Karolinska Institute, Karolinska University Hospital, Department of Infectious Diseases & Virology, Stockholm, Sweden

## Purpose of the study

To identify and predict factors for late HIV diagnosis in Sweden

## Methods

In a prospective study involving 12 Swedish HIV clinics, all newly diagnosed patients, >18 years, are invited to participate to explore what characterize a late presenters (LP), defined by CD4 <350 and/or AIDS diagnosis, from those diagnosed earlier. Demographic and biomedical data are collected as well as HIV-related symptoms and AIDS diagnosis. Four questionnaires are completed; medical history, psychosocial history, general knowledge of HIV and barriers to testing. Enrolment is Oct 2009—Sept 2011. The abstract is a survey of the first 100 patients.

## Summary of results

69% were LPs; 45% had CD4 counts < 200 and/or AIDS; 15% > 500. 70% were immigrants from non-European countries. 57% of these had lived > 1 year in Sweden. This group also had the highest overall risk of being diagnosed late (37/45, 82%) followed by IDU's (3/5, 60%), heterosexuals from the EU (7/12, 58%) and MSM (14/28, 50%). Median age at diagnosis was 35 years for non-Europeans among both LPs and non-LPs. For Europeans it was 49 and 40 years respectively. 29% (18/63, 6 missing data) of late LPs had been investigated or treated for HIV-associated symptoms (STI, hepatitis B or C, seborrheic eczema, penia of the blood, SR elevation or fever of unknown origin > 1 week) including 4 patients with AIDS diagnosis (candidiasis of the oesophagus or wasting) three years previous to HIV diagnosis without having been offered an HIV test. 32% (9/28, 3 missing data) of the non LPs had a history of previous health care contacts for HIV associated symptoms without HIV test performed. 56/100 patients answered the question if they ever had thought of the possibility of being HIV infected. 18% (10/56) responded yes, independent of being a LP or not.

## Conclusions

LPs are even more common in Sweden today than previously known. 2/3 is diagnosed at a stage when treatment already should have started. Of the 70% represented by immigrant population as many as 57% had lived > 1 year in Sweden and earlier testing should have been possible. The group with the lowest risk of late diagnosis was the MSM indicating their higher awareness, although as many as 50% were diagnosed late. As many as 1/3 of all newly diagnosed HIV patients had been in contact with the health care system for HIV associated symptoms without being HIV tested. The awareness of HIV needs to increase both on a population level and among healthcare professionals.

